# Effect of Aerobic Exercise on Lipid Metabolism in Rats With NAFLD

**DOI:** 10.3389/fgene.2022.901827

**Published:** 2022-06-16

**Authors:** Tongxi Zhou, Mengfan Niu, Ruichen Liu, Li Li

**Affiliations:** ^1^ College of Sports and Human Sciences, Harbin Sport University, Harbin, China; ^2^ Graduate Faculty, Harbin Sport University, Harbin, China

**Keywords:** nonalcoholic fatty liver disease, aerobic exercise, lipid metabolism, effect of aerobic exercise, NAFLD

## Abstract

This work aimed to study the intervention effect of exercise on lipid metabolism in NAFLD rats, provide a more scientific experimental basis for exploring and improving the theoretical system of exercise intervention in NAFLD, and further provide a theoretical research basis for clinical treatment of NAFLD. Forty healthy male Sprague Dawley rats were randomly divided into a blank control group (BC,14) and a model group (MO, 26). After 6°weeks of modeling, the MO group was randomly divided into the model control group (MC, 12) and the aerobic exercise group (AE, 12). Platform running intervention in group E was conducted at a slope of 0°, a speed of 15 m/min, 1 h/time, once a day, six times a week, and a day of rest, for 8°weeks in total. After the intervention, the liver tissues of rats were taken for pathological sections, and serum was taken and analyzed for TC, TG, LDL-C, HDL-C, and FFA levels. Under the light microscope, the liver tissue structure of rats in the BC group was complete and clear, the structure of liver lobules was clear and normal, the volume of hepatocytes was uniform, the nucleus was in the middle, and the cytoplasm was red-stained, and no steatosis of hepatocytes was found. The liver of rats in the MC group showed diffuse fatty lesions, disordered structure of hepatic lobules, disordered arrangement of hepatic cords, different sizes of hepatocytes, loose cytoplasm, and diffuse lipid droplets of different sizes in the cytoplasm. The accumulation of liver lipid droplets in the AE group was improved compared with the MC group, the number of fat vacuoles in hepatocytes was significantly reduced, and the degree of liver lipid deposition was reduced. Compared with the BC group, the content of TC, TG, LDL-C, and FFA in the serum of the MC group increased significantly (*p* < 0.01), and the content of HDL-C decreased significantly (*p* < 0.01). Compared with the MC group, the content of TC, TG, LDL-C, and FFA in the serum of the AE group decreased significantly (*p* < 0.01/*p* < 0.05), and the content of HDL-C increased significantly (*p* < 0.01). Therefore, moderate-intensity aerobic exercise has an intervention effect on lipid metabolism in NAFLD rats, which can be used as one of the means to treat NAFLD.

## 1 Introduction

As the most common chronic liver disease (NAFLD), nonalcoholic fatty liver disease has become a huge and increasingly serious public health problem. At this stage, China is in a period of an aging population. It is expected that by the end of 2030, the prevalence of NAFLD in China will increase to 22.2%, and the number of patients will reach 314 million (Estes et al., 2018). At the same time, under the risk factors such as obesity, sedentary lifestyle, and/or susceptible genetic background in the past 15 years, the prevalence of NAFLD in children tends to be higher, and the prevalence of NAFLD in children will reach 13–60%. It has become one of the most common liver diseases in children (Mann et al., 2018), but the pathogenesis of NAFLD has not been fully clarified, and the treatment method is not ideal, which brings a heavy economic and psychological burden to society and families. Therefore, it is necessary to explore the pathogenesis of NAFLD and adopt effective treatment strategies for NAFLD in order to understand the epidemic and reduce the disease burden, which has become one of the essential problems to be solved urgently in front of medical workers.

### 1.1 NAFLD

NAFLD refers to the clinicopathological syndrome diagnosed as primary hepatic steatosis by histological detection or imaging, excluding secondary hepatic fat accumulation factors, such as fatty drugs, genetic diseases, excessive drinking, etc. (Grundy et al., 2005). It is the specific manifestation of metabolic syndrome in the liver. The main pathological change is the imbalance between liver lipid synthesis and oxidation, resulting in abnormal accumulation of triglycerides (TG) and total cholesterol (TC) in hepatocytes and the formation of liver lipid droplets (Donnelly et al., 2005; Malhi and Gores, 2008; Neuschwander-Tetri, 2010). According to the histopathological changes of the liver, the pathological classification of NAFLD ranges from simple hepatic steatosis to Nash with/without fibrosis, to fatty liver cirrhosis and hepatocellular carcinoma (Roeb et al., 2015). Most NAFLD patients only have simple hepatic steatosis, of which about 30% of NAFLD patients will develop into more severe Nash, accompanied by hepatocyte injury and inflammation. Studies have shown that (Vernon et al., 2011) the risk of death from liver disease in nonalcoholic steatohepatitis (NASH) patients with fibrosis and cirrhosis can be increased by 50–80 times, which indicates that the stage of simple hepatic steatosis is not completely benign and has the potential to further develop into hepatic fibrosis. The risk of liver cirrhosis and hepatocellular carcinoma needs to strengthen in prevention and treatment intervention. By the end of 2021, although there are drugs that coexist through the treatment of metabolic syndrome and anti-NAFLD characteristics, no drugs that are completely effective in the treatment of NAFLD have been approved. Lifestyle intervention including diet control and increased physical activity is the first-line treatment of NAFLD (Kraus et al., 2002; Yen et al., 2015).

### 1.2 The Pathogenesis of NAFLD

The potential mechanism of NAFLD occurrence and development is caused by many factors. In the beginning, day and James put forward the “second strike” theory (Qian et al., 2021). The first strike is mainly insulin resistance (IR). The inhibitory effect of insulin on hormone-sensitive lipase is weakened, the ability of peripheral fat decomposition is increased, the content of free fatty acid (FFA) in blood and the intake of FFA by the liver are increased, resulting in lipid deposition in hepatocytes, which increases the sensitivity of liver to damage mediated by the second strike. Based on this, The hepatic uptake of FFA increases, but the oxidative consumption rate of FFA by mitochondria is limited, resulting in the accumulation of FFA in the liver. At the same time, when mitochondria oxidize a large amount of FFA, they will produce excessive reactive oxygen species (ROS) and lipid peroxide, which will cause mitochondrial dysfunction and oxidative stress in the liver, and cause inflammation, fibrosis, and necrosis of hepatocytes; At the same time, the accumulation of excessive FFA, ROS and lipid peroxide in the liver can induce the occurrence of IR, directly affect the metabolic environment of NAFLD, activate various risk factors and innate immune response, recruit various inflammatory factors, and further lead to hepatocyte injury and form a malignant cycle. With the deepening of research, it is found that many factors related to the occurrence and development of NAFLD will affect and promote each other, causing repeated blows to the liver. Therefore, the “second strike” theory has been unable to fully explain some metabolic disorders and molecular mechanisms in the occurrence and development of NAFLD. The “multiple strike” theory proposed by Tilg and Moschen has gradually replaced the “second strike” theory (Buzzetti et al., 2016). The theory of “multiple blows” clarifies that dietary habits, and environmental and genetic factors can jointly affect the changes of IR, oxidative stress, lipid metabolism disorder, adipocyte proliferation and dysfunction, and intestinal microbiota. The occurrence and development of NAFLD result from the interaction between multiple potential pathways and multiple injuries.

As the central regulator of lipid homeostasis, the liver participates in many essential links in lipid metabolism, including lipid uptake and synthesis, lipid processing, storage, oxidative decomposition and output, and their utilization as energy substrates. When the uptake of FFA by the liver exceeds the oxidation and output of FFA, lipids are deposited in hepatocytes in the form of TG, which eventually leads to lipid peroxidation stress and liver injury. Therefore, TG deposition is the key to the formation of NAFLD, and the mechanism of lipid metabolism disorder is not completely clear, which may be related to the following links: increased lipid uptake by the liver, increased *de novo* lipogenesis by the liver, imbalance of lipid oxidation, disorder of lipoprotein synthesis and output, resulting in an imbalance in the synthesis, degradation, and secretion of liver lipid metabolism, resulting in the decrease of TG transport out of hepatocytes. Finally, lipids are abnormally deposited in hepatocytes.

To sum up, liver fat deposition results from unbalanced TG synthesis and oxidation output. Excessive energy intake is the initiating factor, followed by the increase of FFA in the blood, which results in an increase in liver lipid intake. Excessive carbohydrates in the diet can promote an increase in lipid synthesis. Excessive FFA mobilization causes mitochondriaβ. The oxidation ability is impaired, the production of ROS and lipid peroxide is increased, the liver fat deposition and the activation of the liver necrotizing inflammatory response are increased, and the ability of lipid oxidation is decreased. In conclusion, the role of liver lipid deposition in NAFLD and its potential mechanism has important basic theoretical significance, and also provide the molecular biological basis and treatment strategies for the prevention and treatment of the disease.

### 1.3 Progress of Exercise Improving NAFLD

It has become a consensus that a lack of physical exercise can increase the risk of NAFLD in recent years. On the contrary, physical training can reduce the content of liver fat and reduce hepatic steatosis, which is an effective strategy for the treatment of NAFLD. Through the research, it is found that the selection of reasonable exercise mode, the planning of the best exercise intensity, and the formulation of personalized exercise therapy will have a scientific, reasonable, and high curative effect on NAFLD.

#### 1.3.1 Effects of Different Exercise Modes on NAFLD

The selection of NAFLD exercise mode is mainly divided into aerobic exercise and resistance exercise. Both of them can improve the serological indexes and pathological scores of NAFLD. Aerobic exercise is an aerobic metabolic way to consume sugar and fat to provide energy for the body when oxygen is fully supplied to tissues and cells, including jogging, swimming, Tai Chi, and other forms of exercise. In the systematic evaluation study, the relationship between the changes in ALT, AST, IR index, and body mass index and the effects of aerobic exercise training or dietary intervention on NAFLD patients was evaluated. Compared with the observation group, aerobic exercise training plus diet showed a good trend and effect on ALT, AST, IR index, and BMI. In addition, aerobic exercise can regulate and improve the hepatic steatosis and IR status of obese mice by enhancing fat phagocytosis (Li et al., 2021). However, recent studies have shown that the effect of aerobic exercise on the improvement of intrahepatic lipids is not comprehensive, but it can significantly improve IR and lipid droplet size, suggesting that aerobic exercise can delay some aspects of NAFLD, but cannot reduce all metabolic disorders (La Fuente et al., 2019). In addition, in recent years, the prevention and treatment of NAFLD have extended multi-means joint intervention, in order to obtain a better curative effect than simple exercise intervention.

#### 1.3.2 Effect of Different Exercise Intensities on NAFLD

Training intensity may play a key role in enhancing the protective effect of physical exercise on NAFLD. Studies have shown that 12 weeks of moderate-intensity aerobic training can reduce liver fat content in sedentary obese men with NAFLD. Its mechanism may be to regulate lipid metabolism and obesity-related inflammatory state *in vivo* by reducing the gene expression level of lipid synthesis in monocytes (Oh et al., 2017). Low-intensity exercise can also improve NAFLD, but if the load is low, the intervention on liver lipid droplets is not significant. Sufficient load generated during high-intensity exercise can effectively resist and alleviate the formation of liver lipid droplets. Studies have shown that high-intensity training and dietary intervention at 70% VO2max intensity for 6 weeks can improve SOD expression level and T-SOD activity, reduce the degree of lipid peroxidation, and then significantly inhibit the progress of hepatic steatosis (Jiang et al., 2021). In addition, the study of 12-weeks high-intensity exercise training in OLETF rats showed that high-intensity training was effective in improving the liver hardness and restoring Kupffer cell function (Linden et al., 2015). Compared with other exercise intensities, high-intensity exercise is a method of getting twice the result with half the effort because of its short exercise duration and exercise cycle. Still, it needs to be carried out under the scientific guidance of professionals.

To sum up, although the best exercise prescription for the prevention and treatment of NAFLD cannot be fully determined, according to the recommendations of Easl-Easd-Easo clinical practice guide (European Association for the Study of The Liver et al., 2016). The physical activities of the NAFLD population need to reach at least 150–200 Min per week, 3–5 times per week and medium intensity; At the same time, considering that resistance exercise and high-intensity intermittent exercise has a clear adaptive population and require standardized training of professionals, compared with people with chronic metabolic diseases such as NAFLD, the risk of exercise under maximum short-term intensity is high, and the actual operation is difficult, which needs to be further evaluated in practical application; Exercise can be selected according to the individual habit and exercise habit. In addition, in recent years, the prevention and treatment of NAFLD have extended multi-means joint intervention, in order to obtain a better curative effect than simple exercise intervention.

## 2 Materials and Methods

### 2.1 Animals

Male Sprague Dawley rats (200 ± 20 g, 25–35 days of age) provided by Liaoning Changsheng Biotechnology Co., Ltd. (production license No. scxk (Liao) 2020-0001) and raised in the animal laboratory of Heilongjiang University of Chinese Medicine (Harbin, Heilongjiang, China). Rats were housed in groups of 6 animals per plastic cage under controlled conditions of light and temperature (25 ± 3°C) and relative humidity (60–70%). Rats were allowed free access to standard laboratory food and tap water, and to adapt to the laboratory for at least 1 week before the onset of the experimental protocol.

### 2.2 Experimental Design

Forty rats were selected for this study and were fed either a normal diet (blank control group, 14) or a high-fat diet (model group, 26) (2% Cholesterol, 0.5% sodium cholate, 5% sucrose, 10% lard and 82.5% basic feed) for 14 weeks. All animal experiments in this study were approved by the animal laboratory of Heilongjiang University of Chinese Medicine.

After six weeks, two rats in each group were killed and the materials were obtained. To determine whether the molding was successful by observing the results of hematoxylin-eosin (HE) staining of liver tissue. After successful modeling, the model group was randomly divided into the model control group (MC, 12) and the aerobic exercise group (AE, 12).

**FIGURE 1:**
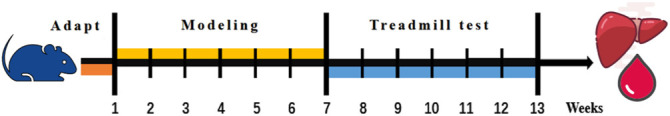
Experimental design.

Referring to T.G.Bedford et al. (Bedford et al., 1979) classic rat treadmill test experiment, the AE group in this experiment adopted the simple speed-up exercise scheme of medium intensity: the treadmill slope was constant 0°, the speed in the first stage was 5 m/min for 5 min, and the speed in the second stage was 15 m/min for 55 min. They trained at the same time every Tuesday to Sunday, and rested on Monday. It lasted for 8 weeks. (Figure 1).

### 2.3 Tissue Processing

24hs after the end of the exercise protocol, rats were injected with chloral hydrate 10% (4 ml/kg) in the abdominal cavity. Blood was harvested, and serum was also collected and stored at −80°C for TC (100,000,220), TG (192061), VDL-C (1,00,020,245), HDL-C (1,00,020,235), and FFA (A042-2-1) analysis. After the completion of blood collection, the liver tissue was dissected quickly and fixed in 4% paraformaldehyde solution for HE staining.

### 2.4 Statistical Analyses

The results were expressed as mean ± standard deviation (x ± s). The data of each group were tested for normal distribution and homogeneity of variance. An independent-sample t-test was used to compare the differences between the two groups. A value of *p* < 0.01 was considered more statistically significant.

## 3 Results

### 3.1 Effect of Liver Histomorphology With High-Fat Diet

**FIGURE 2:**
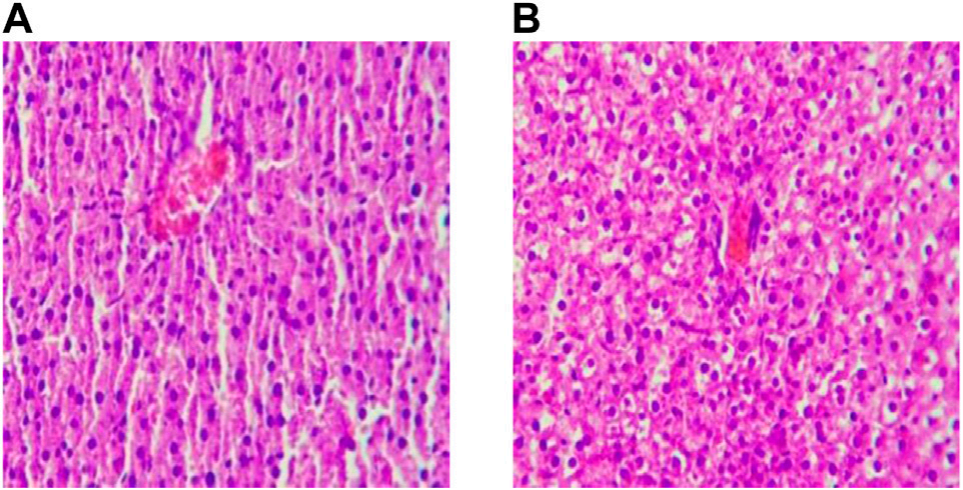
Liver sections after modeling (×200). Note: **(A)** Ordinary Diet Group; **(B)** High-fat Diet Group.

After feeding for six weeks, the hepatocytes of rats in the BC group were arranged regularly. The cytoplasm, nucleus, hepatic cord, and hepatic sinuses were clear. The staining was uniform, and there was no edema and inflammatory cell infiltration in the liver tissue. The hepatocytes of rats in the MO group were grid-shaped, showing obvious steatosis, the obvious proliferation of connective tissue around large blood vessels, and local scattered inflammatory cells gathered in piles, but there was no obvious congestion and expansion of capillaries. The results are consistent with the relevant Literature (Wang and Guang, 2007) report, which indicates that the model establishment of NAFLD in rats was successfully established. (Figure 2).

### 3.2 Effect of Liver Histomorphology With Treadmill Exercise

**FIGURE 3:**
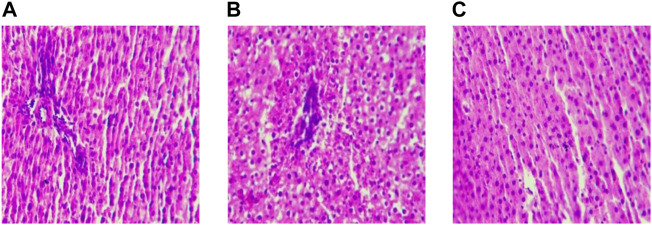
Liver sections after intervention(×200). Note: **(A)** Blank Control Group; **(B)** Model Group; **(C)** Aerobic Exercise Group.

After feeding for eight weeks, the hepatocytes in the MC group still showed a grid shape, the degree of steatosis increased, and the proliferation of connective tissue around large vessels, local inflammatory cell aggregation, and pyknosis of hepatocytes increased significantly. After 8 weeks of aerobic exercise intervention, the liver histopathology of the AE group was improved compared with the MC group. (Figure 3).

### 3.3 Effect of Treadmill Exercise on the Serum Lipid Metabolism Indexes

The levels of TG, TC, LDL-C, and FFA in the serum of the MC group were significantly higher than those of the BC group (*p* < 0.01), while the level of HDL-C was significantly lower than that of the BC group (*p* < 0.01). After aerobic exercise intervention, there was also a very significant difference between the AE group and MC group (*p* < 0.01), and the index results were between the MC group and BC group. The results showed that the lipid deposition was obvious in rats with NAFLD, and aerobic exercise could then improve the serum lipid metabolism imbalance state by regulating the serum lipid metabolism markers. (Table 1).

**TABLE 1:** Serum lipid metabolism indexes (x ± S).

Index group	Blank control group	Model group	Aerobic exercise group
	(n = 12)	(n = 12)	(n = 12)
TG (mmol/L)	0.85 ± 0.10^**^	1.56 ± 0.20	0.92 ± 0.13^**^
TC (mmol/L)	0.94 ± 0.20^**^	1.73 ± 0.10	1.21 ± 0.09^**^
HDL-C (mmol/L)	1.63 ± 0.18^**^	1.01 ± 0.12	1.48 ± 0.22^**^
LDL-C (mmol/L)	0.47 ± 0.08^**^	1.21 ± 0.09	0.78 ± 0.13^**^
FFA (mmol/L)	0.42 ± 0.02**	0.76 ± 0.05	0.46 ± 0.05*

Compared with the model group, *p < 0.05, **p < 0.01.

## Conclusion

NAFLD is a syndrome characterized by a disorder of hepatocyte lipid metabolism (hepatic steatosis) without alcohol and other clear factors of liver damage. Generally speaking, lipid metabolism is regulated through a variety of ways, among which *de novo* lipogenesis is one of the main mechanisms leading to the increase of FFA transport and accumulation in the liver. (Linden et al., 2015) (European Association for the Study of The Liver et al., 2016). This study found that compared with the rats in the BC group, the contents of TC, TG, LDL-C, and FFA, increased HDL-C decreased in the MC group. It indicates that NAFLD rats fed a high-fat diet for a long time caused an imbalance of lipid metabolism, excessive accumulation of hepatocyte lipids, and hepatocyte dysfunction. After aerobic exercise intervention, TC, TC, LDL-C, and FFA in the AE group decreased, HDL-C increased, and the blood lipid index of NAFLD rats improved, which is consistent with the research results of Linden (Linden et al., 2015) and Xu (Xu, 2006). It indicates that regular and appropriate aerobic exercise intervention can inhibit the storage of redundant lipid substances in tissues and cells and improve the disorder of blood lipid metabolism by increasing heat consumption, accelerating lipid oxidation, and reversing lipid deposition, so as to provide a theoretical basis for anti-NAFLD composite targets and joint intervention and provide a reference for the application of aerobic exercise combined with other polysaccharides in the prevention and treatment of NAFLD. Therefore, exercise intervention has unique advantages and certain application prospects.

## Data Availability

The original contributions presented in the study are included in the article/Supplementary Material; further inquiries can be directed to the corresponding author.
